# Special Issue: “Application of Nanotechnology in Regenerative Medicine”

**DOI:** 10.3390/ijms26136393

**Published:** 2025-07-02

**Authors:** Flavia Carton, Manuela Malatesta

**Affiliations:** 1 Department of Neurosciences, Biomedicine and Movement Sciences, University of Verona, 37134 Verona, Italy; manuela.malatesta@univr.it; 2 Center for Medical Sciences (CISMed), University of Trento, 38122 Trento, Italy; 3 Department of Cellular, Computational and Integrative Biology (CIBIO), University of Trento, 38123 Trento, Italy

Regenerative medicine is a relatively young field, born as a convergence of disparate disciplines aimed at restoring or replacing tissues and organs. The term regenerative medicine was coined in 1999 by Dr. William A. Haseltine during a conference held at Lake Como (Italy) [1], but an exhaustive definition accounting for the goals and the multidisciplinary approach of this new field was formulated only in 2007 by Daar and Greenwood [2]. These authors stated, “Regenerative medicine is an interdisciplinary field of research and clinical applications focused on the repair, replacement or regeneration of cells, tissues or organs to restore impaired function resulting from any cause, including congenital defects, disease, trauma and ageing. It uses a combination of several converging technological approaches, both existing and newly emerging, that moves it beyond traditional transplantation and replacement therapies. The approaches often stimulate and support the body’s own self-healing capacity. These approaches may include, but are not limited to, the use of soluble molecules, gene therapy, stem and progenitor cell therapy, tissue engineering and the reprogramming of cell and tissue types”.

Nanotechnology ranks high among these approaches, and especially in the last two decades, its application in regenerative medicine has enormously advanced and proved to be crucial for the development of new materials and tools able to restore and support damaged tissues and organs. Several nanoparticles for drug delivery [3]; carbon-based nanomaterials [4]; nanostructured hydrogels [5,6]; 3D nanofibrous scaffolds [7,8,9,10]; nano-hydroxyapatite-based scaffolds [11]; 2D nanomaterials, such as transition metal dichalcogenides, transition metal oxides, and nanoclay [12,13]; nanobioglass [14]; and even nanogenerators for electrical stimulation [15] have been set up to improve regeneration of various tissues, among which are bone, cartilage, heart, and skin. Graphene-based nanomaterials and electroconductive nanobiomaterials have been developed to promote nerve and heart regeneration [16,17,18]. Exosomes (i.e., nano-sized extracellular vesicles) have been explored as nanoplatforms to deliver therapeutic agents or factors secreted by mesenchymal stem cells to accelerate the regeneration process in diverse organs and tissues [19,20,21,22,23,24,25]. Bacteriophage has attracted attention as a nanotool for applications in wound healing and skin regeneration [26].

In this challenging scientific context, this Special Issue “Application of Nanotechnology in Regenerative Medicine” offers a collection of interesting review articles.

Abdelhamid and co-workers [27] exhaustively reviewed the design strategies at the basis of the fabrication of engineered silica-based biomaterials and their various applications in regenerative medicine. The silica-based nanoconstructs are highly biocompatible, may be prepared with predictable porosity, and proved to modulate cellular behavior at the molecular level. The progress in synthesis and functionalization of nanoengineered silica allowed manufacturing a new generation of versatile nanomaterials designed for targeted drug delivery and the construction of biomimetic scaffolds integrating stem cell therapy. Thanks to these peculiarities, nanoengineered silica-based biomaterials have the potential to enhance tissue regeneration by improving the drug’s therapeutic efficacy and modulating stem cell behavior. Interestingly, these silica-based nanoconstructs are also useful for non-invasive diagnostics and the monitoring of treatments through advanced imaging techniques.

Biominerals are inorganic solids that a wide variety of organisms naturally produce to harden tissues. The article by Kim et al. [28] reviewed the properties and the synthesis procedures of biominerals and their composite materials and examined the role of these constructs in regenerative medicine. Composite materials are designed to mimic the tissue’s morphological, mechanical, and biochemical features, which is crucial for effectively promoting regeneration. Polymers or metallic materials are conventionally used as scaffolds to provide a 3D substrate for cell adhesion and proliferation and facilitate regeneration. However, they are often inadequate from a mechanical point of view or may be potentially toxic. Incorporating biominerals into these materials allows enhancing their strength, durability, and biocompatibility while improving the cell interaction and bond to the implant by mimicking the natural micro-environment. Biomineral composites also proved to have wound-healing properties and exert an antimicrobial effect, and they can be engineered as efficient targeted drug carriers with minimized side effects.

Carton and Malatesta [29] provided an overview of the uses of innovative hyaluronic acid-based nanoconstructs in regenerative medicine. As a main component of the extracellular matrix in almost all body tissues and fluids of vertebrates, hyaluronic acid is crucial for tissue hydration and osmotic pressure maintenance; moreover, it plays a key role in several biological processes, e.g., cell growth, differentiation and motility, angiogenesis, and inflammation. Hyaluronic acid is widely used in regenerative medicine due to its physiological effects in tissue repair, and especially in recent years, nanomedical formulations containing hyaluronic acid have been developed to improve this process. In particular, hyaluronic acid has been used to create multifunctional hydrogels containing nanoconstructs as structural and functional constituents; these hydrogels are presently applied in orthopedics, dermatology, and neurology. In the years to come, the progress of nanotechnological engineering will allow the design of innovative treatments based on hyaluronic acid nanodevices suitable not only for regenerative medicine but also for precision and personalized medicine and theranostics.

Sandoval and co-workers [30] reviewed the application of cerium oxide nanoparticles in non-alcoholic fatty liver disease. Thanks to their antioxidant capabilities, cerium oxide nanoparticles were investigated for their effectiveness through in vivo and in vitro studies from 2012 to 2023 from the MEDLINE, EMBASE, Scopus, and Web of Science databases. It was found that the administration of cerium oxide nanoparticles is a promising treatment option for liver illnesses, as a decrease in steatosis, necrosis, and the production of reactive oxygen species was observed in non-alcoholic fatty liver disease. In the attempt to elucidate the etiology of this disease, future studies are, however, needed to overcome the present limitations due to the different durations and models used in the studies considered.

Micro- and nanostructured biocompatible coatings have been produced to limit corrosion and hydrogen release from orthopedic devices made of magnesium alloy, which are receiving increasing interest because of their mechanical properties and bioresorption potential. In their systematic review based on four databases (PubMed^®^, Embase, Web of Science™, and ScienceDirect^®^), Giavaresi and co-workers [31] reviewed the approaches, coating procedures and strategies aimed at improving biocompatibility, osteogenic properties, and osteointegration of magnesium alloy devices, whose utilization in orthopedics is currently hampered by their rapid degradation. A comprehensive analysis of 40 preclinical studies in vitro and in vivo was provided, demonstrating the continuous research progress in the surface modifications of magnesium alloys by micro- and nanocoatings with organic or inorganic nanocomposites in the attempt to improve the long-term mechanical resistance to loading and support osteointegration, thus promoting functional bone regeneration.

The prominent role played by nanotechnology and biomedical engineering in medicine was underlined by Silva et al. [32]. Significant impacts on human health were generated by the intersection of these disciplines, as demonstrated by the advances in diagnostics and imaging, the development of nanobiosensors, the application of nanofibers and nanowires as biocompatible scaffolds in tissue engineering, and the efficacy of nanoconstructs in controlled drug release and regenerative therapies. The authors, however, underlined that challenges still are to be overcome to fully exploit the potential of nanotechnology in regenerative medicine and suggested that a multidisciplinary perspective should be taken to assess the sustainable use and biocompatibility of nanomaterials, as well as their bioavailability and interaction with cells, tissues, and organs.

The future of nanotechnology in regenerative medicine holds immense potential despite present limits, as has been underlined by all the authors of this Special Issue. The interdisciplinary collaboration between biologists, material scientists, experts in computer science, and clinicians will be crucial to entirely understand the complex interactions between the nanosystems and the biological environment. No doubt, integrating artificial intelligence into the process of nanoconstruct design will be helpful in rapidly developing safe and effective regenerative nanotools.

There is a lot of work still to be completed, but the prospects are wide and exciting.
